# Patient-preferred outcomes in patients with vestibular schwannoma: a qualitative content analysis of symptoms, side effects and their impact on health-related quality of life

**DOI:** 10.1007/s11136-023-03433-x

**Published:** 2023-05-31

**Authors:** Ineke M. J. Pruijn, Phylisha van Heemskerken, Henricus P. M. Kunst, Marcia Tummers, Wietske Kievit

**Affiliations:** 1 Department of Otorhinolaryngology, Radboud University Medical Center, Radboud Institute for Health Sciences, P.O. Box 9101, 6525 EX Nijmegen, The Netherlands; 2 Dutch Academic Alliance Skull Base Pathology, Radboud University Medical Center, Maastricht University Medical Center, Nijmegen/Maastricht, The Netherlands; 3 Department of Health Evidence, Radboud University Medical Center, Radboud Institute for Health Sciences, Nijmegen, The Netherlands; 4 Department of Otorhinolaryngology, Maastricht University Medical Center, Maastricht, The Netherlands

**Keywords:** Vestibular schwannoma, Acoustic neuroma, Health-related quality of life (HRQoL), Patient-preferred outcome, Patient-centred care

## Abstract

**Purpose:**

During counseling and management of patients with vestibular schwannoma (VS), the emphasis is shifting from tumour control and nerve preservation towards maintaining or improving health-related quality of life (HRQoL). Understanding the patients’ perspective and impact of VS is, therefore, of utmost importance. The current study aimed to identify treatment outcomes preferred by patients and to explore the patient-reported VS symptoms and management-related side effects and their impact on HRQoL.

**Methods:**

Patients with VS were contacted through the Dutch VS association Stichting Hoormij and questioned using a semi-structured, cross-sectional online survey. Patients were asked to report and rank symptoms and side effects, with their impact on HRQoL and frequency of occurrence. Results were structured through qualitative content analysis. Coded symptoms, side effects, impacts, frequencies, and patient-preferred outcomes were analysed and summarized with descriptive statistics.

**Results:**

Of the 231 respondents, 71% were actively treated. Hearing (symptoms vs. side effects: 78.8% vs. 63.6%), balance (62.3%; 48.8%), and energy issues (33.8%; 32.6%) were the most frequently mentioned symptoms and management-related side effects. Fatigue, deafness, headaches, and hearing loss had the highest impact on HRQoL. The majority of patients identified hearing preservation (61%), balance preservation (38.5%), and reduced tinnitus (34.6%) to be the patient-preferred outcomes.

**Conclusion:**

This qualitative study demonstrates that in this population many patients with VS encounter participation difficulties in their daily physical and social activities and value hearing and balance preservation, reduced tinnitus, and restored energy as preferred outcomes as they are hampered by symptoms and side effects related to hearing, balance, and energy. Healthcare professionals should consider these key points and use these and the patient-preferred outcomes in consultation, shared decision making, treatment, and follow-up to optimize patient-centred care.

**Supplementary Information:**

The online version contains supplementary material available at 10.1007/s11136-023-03433-x.

## Plain English summary

Vestibular schwannomas (VS) are rare benign tumours of the hearing nerve. They typically cause hearing loss, tinnitus and balance disorders, but also headaches, a lack of energy and anxiety. Management of vestibular schwannomas is aimed at maintaining health-related quality of life (HRQoL) while preventing symptoms to get worse as a result of the tumour or treatment. In this study, we have explored the patients’ perspective on VS, its impact on HRQoL and the outcomes that patients consider most important when measuring and evaluating their disease-burden (patient-preferred outcomes). This study indicates that many patients with VS encounter participation difficulties in their daily physical and social activities and are mostly hampered by symptoms and side effects related to hearing, balance and energy. Treatment outcomes that patients place most importance on are hearing preservation, balance preservation and reduced tinnitus. Healthcare professionals should consider these key points and use these and the patient-preferred outcomes in consultation, treatment and follow-up to optimize patient-centred care.

## Introduction

Vestibular schwannoma (VS) is a rare, slow-growing, benign tumour originating from the Schwann cells surrounding the eighth cranial nerve. It is the most common cerebellopontine angle tumour, with an incidence of about 34 per 1,000,000 [1] and a median age at diagnosis of 50 years [2]. VS is most commonly characterized by unilateral hearing loss, tinnitus and dizziness but may also lead to a lack of energy, anxiety, headaches and balance problems. Current treatment for patients with VS is generally aimed at clinical outcomes such as tumour control, hearing and facial nerve preservation [3–7]. Treatment options include observation with serial imaging in a wait and scan protocol, stereotactic radiosurgery and microsurgery [3, 7]. Unfortunately, neither one of these management strategies can improve the experienced hearing loss, tinnitus or balance problems. Additionally, all treatment options have a risk of deteriorating hearing, balance or facial nerve function. As VS is a benign tumour and usually not a life-threatening disease, patients will face the associated symptoms and/or management-related side effects (hereafter shortened to side effects) for the rest of their lives [8]. Consequently, VS is considered a chronic disease with a lifelong impact on the patient’s health-related quality of life (HRQoL). As of such, especially in patients with the chronic sequelae of VS, HRQoL is emerging as an increasingly important outcome in decision for treatment and treatment evaluation [9, 10].

QoL is characterized by the World Health Organization as: ‘The individual’s perception of their position in life in the context of the culture and value system in which they live’, and is influenced by six domains [11]. Four of these domains (physical, psychological, functional, and social) imply the influence of health on the QoL, also defined as the HRQoL [12]. Covering physical as well as mental needs, HRQoL is an important concept in disease perception, disease assessment and decision making [13]. HRQOL is driven by patient preferences, recognizing patients may prioritize and prefer additional or other treatment outcomes than clinicians, which are shaped by the patient’s beliefs, perspective, expectations and goals for health and life [14].

In order to incorporate the patient’s HRQoL in management strategy decision, HRQoL should be periodically measured, or at least the factors that impact HRQoL. HRQoL in patients with VS has been assessed by several researchers, mainly using generic questionnaires such as the 36-Item Short Form Health Survey (SF-36) and Glasgow Benefit Inventory (GBI) [5, 13, 15–25], and less frequently, with the disease-specific Penn Acoustic Neuroma Quality-of-Life (PANQOL) questionnaire [3, 4, 26–28]. Although these questionnaires measure HRQoL, they all restrict patients to pre-determined domains and answer options. Likewise, patient-preferred outcomes in patients with VS are highly understudied with only one study limited to prefilled possible impairments [29].

Understanding the patients’ perspective on VS, its impact on HRQoL and the patient-preferred outcomes (what outcomes matter most to patients) is important and essential in the process of shared decision making and, thus, requires deeper investigation. Taking into account, the considerable morbidity and impact on HRQoL with which VSs and their treatments are associated [30], insights in the patient perspective could be used during consultations, in counselling, and decision making. Therefore, the current study aimed to identify patient-preferred outcomes and to explore the patient-reported disease symptoms and management-related side effects in patients with VS in order to understand how these affect their HRQoL and daily life.

The expected outcomes of this study are a rich understanding of the experiences of patients with vestibular schwannoma and the factors that influence their HRQoL and daily life activities. We think these will mainly concern restrictions in societal participation and employment affecting HRQoL rather than the classical management outcomes such as tumour control and functional nerve preservation influencing HRQoL. The study will contribute to a deeper understanding of the challenges faced by patients with VS and inform the development of patient-centred care strategies that address their unique needs and preferences.

## Methods

### Study design and participants

This cross-sectional qualitative study used a phenomenology approach and was carried out through an online self-report survey in a cohort of Dutch patients with VS. The online survey was open for enrolment for two weeks in April 2020. Printed surveys were provided on request. In cooperation with Stichting Hoormij, the Dutch patient organization for patients with hearing and balance problems, the online survey was distributed among patients with VS. To enhance trustworthiness, purposeful sampling was used, reaching out to all people with VS in the Netherlands, our group of interest. Participants were not recruited via their hospitals, though this may seem convenient, since this may raise ethical, legal and practical concerns related to patient privacy and selection bias as participants may feel obliged to participate on request of their hospital or healthcare provider, compromising the voluntary nature of participation. Stichting Hoormij contacted 332 patients with VS through email for participation and spread the call on their website, through their bi-monthly newsletter (reach of approximately 20,000 hearing/balance impaired patients) and their social media channels. The message on social media was picked up by patients with VS and spread through their VS Facebook community groups to reach as many participants with VS as possible. Patients were included if they had an unilateral VS (self-reported) and were able to fill in the questionnaire. All participants completed the questionnaire without assistance. Participation was voluntary and anonymously without financial compensation to enhance trustworthiness as incentives may lead to bias in participant motivation, a distortion of participant responses (modifying answers to qualify for the incentive) and ethical concerns related to vulnerable populations where individuals may feel compelled to participate in research due to their vulnerable circumstances or financial need, rather than making an informed decision based on their own autonomy and understanding.

### Ethical considerations

This study was conducted according to the ethical standards of the Radboudumc Regional Review Board (Nijmegen, the Netherlands) and the 1964 Helsinki declaration and its later amendments or comparable ethical standards. All study participants were informed about the voluntary and anonymous participation in the study and provided informed consent for participation and publishing of the results for scientific purposes.

### Questionnaire

We developed a semi-structured questionnaire containing open-ended and Likert-scale questions to obtain a wide variety of patient expressions on perceived symptoms, side effects and their impact on HRQoL. The questionnaire was developed by two researchers (IP and PH) and assessed by two experts in the team (WK and MT) on face and content validity. The questionnaire was pilot tested by non-medical volunteers to enhance and test the comprehensibility and adjusted accordingly.

After providing informed consent, patients were guided through the first questions on demographics. Information on tumour size or classification was not expected to be accurately known and, therefore, not requested. The second part of the questionnaire assessed the self-reported VS-related symptoms and associated restrictions in daily life activities. If patients were treated (stereotactic radiosurgery, microsurgery or both), they were asked to recall the VS-related symptoms and restrictions before treatment. Open-ended questions were used to inquire VS-related symptoms, followed by a ranking of 5 of the most severe symptoms. For every symptom, the frequency of occurrence was assigned on a 4-point Likert scale (rarely; sometimes; often or continuously), and the impact on HRQoL was measured by assigning a number from 1 to 10 (1 being a low impact on HRQoL; 10 being a high impact on HRQoL). Additionally, we assessed whether (closed questions) and how (open-ended questions) these symptoms had an impact on the daily life activities (physically and socio-emotionally). The third part of the questionnaire focused on self-reported side effects. Patients were asked to mention and rank the side effects and determine their frequency of occurrence and impact on HRQoL and daily life activities. The last part of the questionnaire enquired patient-preferred outcomes through open-ended questions, assessing what specific or additional outcomes or treatment expectations patients would prioritize most relevant in managing their disease. Lastly, patients had the opportunity to leave additional remarks or recommendations at the end of the form. Except for the additional remarks, answering all questions was mandatory and proceeding without answering the question or rating the symptoms and side effects (if applicable) was impossible.

### Data analysis

Qualitative inductive content analysis was used to code the VS-related symptoms and side effects, restrictions in daily life activities and patient-preferred outcomes from the open-ended questions. Concepts derived from open-ended questions were tagged independently by two authors (IP and PH), to increase the comprehensibility and provide sound interpretation of the data, and summarized using inductive coding with ATLAS.ti (version 8.4.20 ATLAS.ti Scientific Software Development GmbH, Berlin, Germany) until no new codes emerged and data saturation was reached [31]. During the process, codes were checked on consistency, discussed and adjusted by mutual agreement and categorized into an uniform codebook. Using the codebook, IP and PH separately coded all the concepts derived from the open-ended questions and rankings, which were used to compare and assess the inter-rater agreement. Codes were discussed and verified throughout this process with the team (IP, PH, WK and MT). Patients were able to express multiple symptoms within one category. The number of symptom expressions, therefore, occasionally outnumbered the number of patients and was calculated as the percentage of total citations within one category. If patients mentioned multiple symptoms within one ranking number while only asked for one per ranking, we consequently counted the first reported symptom with its impact and frequency number in any of these cases. Quotations of participants were used to indicate the trustworthiness of results and show conformability [32]. The impact of symptoms and side effects on HRQoL and daily life activities mentioned by ≥ 10% of the participants was considered clinically relevant and sufficient for generalizability. Categories were ranked to determine the most frequent mentioned symptoms and side effects. All demographic, clinical, and rating data were entered into Excel and analysed with descriptive statistics. Data were summarized using frequencies (in percentages), and the impact on HRQoL specifically was presented as the mean of all impact scores for each symptom within a category with standard deviation (SD).

## Results

In total, 237 surveys were completed throughout the multiple platforms (e.g. Stichting Hoormij website and email, Facebook). After exclusion of patients with neurofibromatosis type 2 (*n* = 3), meningiomas (*n* = 1), and removal of duplicates (*n* = 2), 231 completed surveys remained eligible for analysis. The demographics of these patients are presented in Table 1. Patients had a mean age of 58 years (SD 11.5, range 24–86 years), and a slight majority was female (*n* = 144; 62.3%). Mean time since VS diagnosis was 8.3 years (SD 7.5). Most of the patients did not report any comorbidities (*n* = 153, 66.2%). Those patients with comorbidities (*n* = 78, 33.8%) mainly described a variety of diseases such as migraine, glaucoma, and Graves’ disease. In total, 164 patients (71%) were actively treated (stereotactic radiosurgery, microsurgery or a combination of both) for their VS, with a mean time since treatment of 8.4 years (SD 8.2). The other 67 patients (29%) were observed in a wait and scan protocol with serial imaging.

**Table 1:** Demographics

	*N* = 231
**Mean age, years (SD; range)**	58 (11.5; 24–86)
**Sex**	
Female, *n* (%)	144 (62.3%)
**Comorbidities, n (%)**	
None	153 (66.2%)
Other^a^	29 (12.6%)
Chronic pulmonary disease	15 (6.5%)
Hypertension	14 (6.1%)
**Management, n (%)**	
Wait and scan	67 (29.0%)
Stereotactic radiosurgery	61 (26.4%)
Microsurgery	78 (33.8%)
Microsurgery and stereotactic radiosurgery	25 (10.8%)
**Mean time since diagnosis, years (SD; range)**	8.3 (7.5; 0–42)
**Mean time since treatment, years (SD; range)**	8.4 (8.2; 0–42)

*SD* standard deviation, *n* number

^a^Migraine, glaucoma, Graves’ disease etc.

### Patient-preferred outcomes

**Fig. 1:**
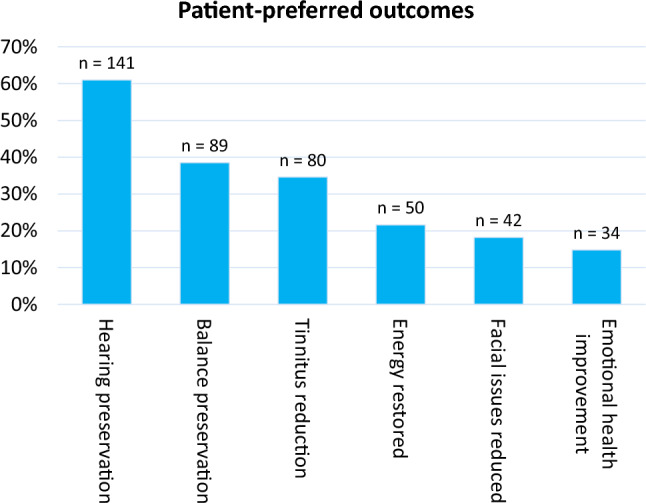
Patient-preferred outcomes. *N* = 231 patients with a total of 520 expressions. *QoL* quality of life. Outcomes preferred by <10% of the participants are not shown

All 231 patients reported at least one patient-preferred outcome, with 520 expressions in total. Patients emphasized the importance of hearing preservation as a preferred outcome (*n* = 141, 61%) (Fig. 1, Supplementary Table 1): *I would be willing to do a lot, just to be able to hear normal again.* Second, patients preferred their balance-related problems (*n* = 89, 38.5%) to be solved. One patient wrote that he wished to *be able to walk again (balance)*, whereas another patient mentioned *that life would be much easier with restored balance*. Third, 80 patients (34.6%) reported that they wished the treatment would decrease or diminish tinnitus. Accordingly, several patients also mentioned that a decreased tinnitus might positively influence other symptoms. This was followed by restored energy (*n* = 50, 21.6%) and solving face-related issues (*n* = 42, 18.2%). Patients mentioned that treatment, in particular, should preserve facial nerve function (*n* = 34 of 45 symptom expressions, 75.6%).

### VS-related symptoms and impact on HRQoL and daily life

After coding the ranked symptoms of all patients (*n* = 231), we identified ten categories, as shown in Table 2. Except for the category ‘tinnitus’ and ‘other’, all categories were further divided into subcategories. Patients were able to express multiple symptoms within one category, explaining the higher number of symptom expressions compared to the number of patients. The most frequently mentioned VS-related symptoms were related to ‘hearing’ (*n* = 182, 78.8%), ‘balance’ (*n* = 144, 62.3%), ‘tinnitus’ (*n* = 118, 51.1%) and ‘energy’ (*n* = 78, 33.8%). Within the category ‘hearing’ (*n* = 182; 191 symptom expressions), patients mentioned being completely deaf (*n* = 94, 49.2%) or having a one-sided hearing loss (*n* = 79, 41.4%). It interfered patients in having conversations: *due to this, I have more difficulties with conversations, on the phone, in meetings, *etc. Most patients reported that both deafness and hearing loss were a continuous burden (respectively, 68.1% and 58.2%). In the ‘balance’ category (*n* = 144; 155 symptom expressions), the majority reported imbalance issues (*n* = 116; 74.8%). However, for 44.8% of the patients who reported this issue, it appeared to bother them merely sometimes. In contrast, dizziness—reported by 33 patients (21.3%)—seemed to affect the patients’ life often (51.5%). More than half of all 231 patients reported they experienced ‘tinnitus’ (*n* = 118, 51.1%), which they described as a *continuous hum*, *beep*, or *bothersome noise*. Most of them reported being continuously burdened by ‘tinnitus’ (63.6%). Lastly, within the category ‘energy’ (*n* = 78), 65 patients (83.3%) experienced fatigue-related symptoms, of which several reported they *get tired faster*, bothering the majority of the group often (58.5%). Apart from the rarely to continuously burden of VS-related symptoms, the highest mean impact on HRQoL was experienced by patients suffering from fatigue (7.3; SD 1.6), deafness (7.2; SD 1.9) and headaches (7; SD 2.2).

**Table 2:** Experienced VS-related symptoms, their impact and frequencies

Categories	*N* = 231						
	Participants, n (% of total)	Symptom expressions, *n* (% of total citations)	Mean impact on HRQoL (SD)	Frequency (%)			
				Continuous	Often	Sometimes	Rarely
Hearing	182 (78.8%)	191					
Deafness		94 (49.2%)	7.2 (1.9)	68.1	22.3	9.6	0.0
Hearing loss		79 (41.4%)	6.8 (2.2)	58.2	30.4	7.6	3.8
Hyperacusis		9 (4.7%)	7.9 (1.2)	44.4	44.4	11.1	0.0
Sound localization		7 (3.7%)	6.7 (2.6)	28.6	57.1	14.3	0.0
Other		2 (1.0%)	5 (NA)	0.0	50.0	50.0	0.0
Balance	144 (62.3%)	155					
Imbalance		116 (74.8%)	5.9 (2.3)	22.4	31.0	44.8	1.7
Dizziness (vertigo)		33 (21.3%)	6.8 (1.9)	27.3	51.5	21.2	0.0
Light-headed		4 (2.6%)	5 (2.6)	0.0	50	50	0.0
Nausea		1 (0.6%)	5 (NA)	0.0	0.0	100	0.0
Drunk-walking		1 (0.6%)	5 (NA)	0.0	0.0	100	0.0
Tinnitus	118 (51%)	118	6.6 (2.2)	63.6	21.2	10.2	1.7
Energy	78 (33.8%)	78					
Fatigue		65 (83.3%)	7.3 (1.6)	27.7	58.5	13.8	0.0
Other		13 (16.7%)	8.2 (1.0)	53.8	38.5	7.7	0.0
Cognitive impairment	47 (20.3%)	56					
Concentration		20 (35.7%)	7.4 (1.5)	15	65	20	0.0
Overstimulated/chaotic		15 (26.8%)	7.5 (1.7)	26.7	53.3	20	0.0
Sleeping		8 (14.3%)	6.8 (1.5)	25	37.5	25	12.5
Memory impairment		7 (12.5%)	6.1 (2.1)	0.0	57.1	42.9	0.0
Other		6 (10.7%)	7.2 (3.3)	33.3	33.3	16.7	16.7
Face	47 (20.3%)	54					
Facial nerve		21 (38.9%)	6.9 (2.7)	47.6	47.6	5	0.0
Trigeminal nerve		17 (31.5%)	5.7 (2.9)	47.1	23.5	29.0	0.0
Eyeproblems		12 (22.2%)	6.5 (2.5)	25.0	33.3	25.0	16.7
Smell and taste		4 (7.4%)	4.8 (2.8)	75.0	25.0	0.0	0.0
Psychological impairment	40 (17.3%)	52					
Anxiety		17 (32.7%)	5.5 (2.1)	17.6	23.5	41.2	17.6
Insecurity		14 (26.9%)	6.1 (1.6)	0.0	50	42.9	7.1
Other		10 (19.2%)	5.8 (2.3)	20	30	40	10
Stress		5 (9.6%)	7.8 (1.3)	20	80	0.0	0.0
Sadness/depression		3 (5.8%)	6.0 (2.6)	33.3	33.3	33.3	0.0
Emotional		2 (3.8%)	5.5 (NA)	0.0	100	0.0	0.0
Frustration and tension		1 (1.9%)	7.0 (NA)	0.0	0.0	100	0.0
Socioeconomical	33 (14.3%)	40					
Social interaction		21 (52.5%)	7.9 (1.6)	33.3	57.1	9.5	0.0
Avoiding busy places		7 (17.5%)	6.4 (1.6)	14.3	57.1	28.6	0.0
Other		5 (12.5%)	6.8 (1.6)	40	40	20	0.0
Leisure time (hobby, sports)		4 (10%)	7.8 (1.3)	25.0	50	50	0.0
Job (loss/not functioning)		3 (7.5%)	9 (1.7)	66.7	0.0	33.3	0.0
Pain	32 (13.9%)	33					
Headache		25 (75.8%)	7 (2.2)	16.0	60	20	4.0
Earache		3 (9.1%)	4 (3.5)	0.0	0.0	0.0	0.0
Not specified		3 (9.1%)	8 (2.0)	33.3	66.7	0.0	0.0
Neck pain		2 (6.1%)	8 (NA)	50.0	50.0	0.0	0.0
Other	11 (4.8%)	11	6.6 (2.8)	36.4	27.3	27.3	9.1

*VS* vestibular schwannoma, *n* number, HRQoL health-related quality of life, *SD* standard deviation, *NA* not applicable

In total, 172 of the 231 (74.5%) patients mentioned that their VS-related symptoms impacted their daily lives (Table 3). Concepts reported by these patients resulted in 10 identified categories. Patients mainly encountered problems during social engagements (*n* = 136, 79.1%). Several occasions in which symptoms burdened them were visiting restaurants and (birthday) parties or other (group) activities: *I am only going out now when we are in a small group.* For some, family life was also affected, either in activities: *playing with my children, because of my dizziness*; or in their communication: *In case of background noise, my husband must repeat himself very often, because I cannot hear what he is saying. This is annoying.* Patients also felt hindered during group conversations mentioning they preferred one-to-one conversations during, for instance, parties: *I can only focus on one person; therefore, I am missing many conversations*. Second, patients experienced difficulties in their work and tasks (*n* = 107, 62.2%), ranging from difficulties in meetings to adaptations in their tasks or quitting their jobs: *I am a teacher and provided lessons to big classes, which has become impossible due to deafness and energy disbalance. Currently, I am teaching one-to-one and coaching other teachers*. Other issues at work related to noises *colleagues have to turn off their radio when I am working*, or a lack of concentration. Third, patients reported to avoid busy places (*n* = 83, 48.3%) as the background noise even affected other symptoms *when it is too noisy, it will result in tinnitus*.

**Table 3:** Impact of VS-related symptoms on daily life

Categories	*N* = 172	
	Participants, *n* (%)	Symptom expressions (% of total citations)
Social engagements	136 (79.1%)	208
Social contacts/activities		111 (53.4%)
Group conversations		97 (46.6%)
Work and tasks	107 (62.2%)	140
Job		83 (59.3%)
Domestic chores		36 (25.7%)
Other		8 (5.7%)
Gardening		7 (5.0%)
Education		6 (4.3%)
(Avoiding) busy place	83 (48.3%)	83
Sports	58 (33.7%)	62
General, sports		47 (75.8%)
Hiking		9 (14.5%)
Running		3 (4.8%)
Mountain-biking		2 (3.2%)
Spinning		1 (1.6%)
Leisure activities	46 (26.7%)	52
Enjoying music		30 (57.7%)
Hobby		22 (42.3%)
Cultural activities	41 (23.8%)	49
Theatre/cinema		30 (61.2%)
Concerts/festivals		16 (32.7%)
Travelling		2 (4.1%)
Other		1 (2.0%)
Traffic participation	34 (20%)	43
Cycling		25 (58.1%)
Walking		7 (16.3%)
Car driving		5 (11.6%)
Other		3 (7.0%)
Motor-cycling		2 (4.7%)
Avoidence of public places		1 (2.3%)
Other	27 (15.7%)	27
Personal care	2 (1.2%)	2
Emotional expressions	2 (1.2%)	2

### Side effects and impact on HRQoL and daily life

One category was added to the existing symptom categories for the codebook of the side effects: ‘post-treatment complications’ (e.g. wound infection and thrombosis) (Supplementary Table 2). Side effects were mainly reported by treated patients (*n* = 125) compared to patients in a wait and scan protocol (*n* = 4). Like VS-related symptoms, most patients who experienced side effects (total *n* = 129) reported that their side effects mainly pertained to ‘hearing’ (n = 82, 63.6%, 85 symptom expressions) though many patients reported that they experienced deafness (*n* = 57, 67.1%). Some of them specified that it occurred after treatment: I *did not have any hearing loss before treatment, after that, bad hearing.* and *Completely deaf on the right side since the operation.* Many patients facing side effects also reported to have ‘balance issues’ (*n* = 63, 48.8%) after treatment. On the contrary, patients encountering side effects experienced ‘face’-related problems more often than untreated patients (*n* = 51, 39.5%, 61 symptom expressions). Most of them related to a (transient) palsy or paralysis of the facial nerve (*n* = 35, 57.4%). Patients reported *face spasms* or *sometimes having a crooked mouth*, the majority reporting that it burdened them continuously (54.3%).

The side effects with the highest impact on HRQoL were deafness (7.5; SD 2.2), fatigue (7.5; SD 1.8) and hearing loss (7.4; SD 1.5). Comparable to VS-related symptoms, patients experiencing side effects encountered problems in their daily lives during social engagements (*n* = 72, 86.7%), had difficulties in their work and tasks (*n* = 50, 60.2%), and avoided busy places (*n* = 47, 56.6%) (Supplementary Table 3).

## Discussion

This qualitative study aimed to assess patient-preferred outcomes in patients with VS. Of the 231 respondents in this study population, the majority was actively treated (71%). Patients mainly prioritize preservation of hearing and balance, tinnitus reduction and restoring energy as patient-preferred outcomes. Second, patient-perceived symptoms and management-related side effects and their impact on HRQoL in patients with VS were explored. For both symptoms and side effects, patients mentioned reduced hearing, reduced balance and energy loss as most frequent issues. Overall, patients were most frequently affected in the socio-economic aspects of life (social engagements, work and tasks) and avoided busy environments.

Patient-preferred outcomes in patients with VS are highly understudied. A systematic PubMed search combining all search terms for VS with a standardized search query on patient preferences for treatment outcomes [33] retrieved one article for full-text assessment [29]. In that study, 576 VS patients ranked prefilled impairments on a scale of 1 (not too bad) to 5 (unbearable). Complete loss of hearing on both sides was ranked highest among the patient-preferred outcomes. Correspondingly, hearing preservation, in our open-ended questionnaire, turned out to be the most favoured patient-preferred outcome. Furthermore, patients in our study ranked balance preservation, tinnitus reduction and restored energy as important patient-preferred outcomes, but these may differ between patients and need to be enquired individually. Clinicians may, however, use and incorporate these preferred outcomes to improve counselling, better support their patients and improve the patients’ satisfaction [33, 34].

Patients often cited hearing and communication problems altogether with problems in their social interactions (Table 3, Supplementary Table 3). Deafness and hearing loss had a high impact on HRQoL and because of their symptoms or side effects patients reported to be hampered especially during social engagements. Although several studies showed that hearing impairment was less substantial in patients’ HRQoL, which might be attributed to a gradual adaptation to the hearing impairment over the years [27, 35], Breivik et al. found that VS patients had significant worsening of social functioning due to hearing loss [36]. Additionally, Carlson et al. developed and validated a new disease-specific QoL instrument for patients with VS as they felt domains that frequently impact well-being such as social engagement and occupational limitations were missing in the PANQOL [37].

Energy-related issues, especially fatigue, had a high impact on HRQoL and restored energy is among the most reported patient-preferred outcomes [9]. Unfortunately, studies specifically on fatigue or a lack of energy in VS patients are scarce. Despite this, Dhayalan et al. recently showed that patients with VS significantly suffer from fatigue complaints. They found a strong relationship between fatigue and other complaints such as depression and anxiety as also vertigo, one of the hallmarks of VS [38].

According to the patients in our study, balance issues—and especially vertigo—did not have the highest impact on HRQoL. An explanation for this phenomenon might be that symptoms like dizziness or vertigo are episodic and transient while hearing loss, deafness and facial weakness may be continuously present. Patients, however, prioritized vertigo among balance as the second highest patient-preferred outcome (Supplementary Table 1), which is in line with the many studies that showed a strong negative association between vertigo and HRQoL [30, 35, 36, 39] and ongoing dizziness being associated with a deterioration of long-term HRQoL in patients with VS [27].

Regarding side effects, facial nerve issues were often mentioned, and deafness was more frequent in treated than observed patients. This might be explained by the fact that treated patients often have larger tumours [40]. Another explanation is that treatment itself, either surgical removal or radiation therapy, might have caused nerve damage and facial dysfunction. Although not pointed out by our results as this was not explicitly investigated, Blom et al*.* showed that facial issues or paresis are debit to a lower HRQoL [41]. However, permanent facial weakness after surgery or radiosurgery is reported to be less than 10% [7, 42]. Similar to facial dysfunctions, headaches (both transient and permanent) are a well-known consequence of VS surgery and more commonly associated with the retrosigmoid approach compared to those treated by the translabyrinthine and middle fossa approaches [43]. Headaches in general, however, though the causal relation with VS is not exactly clear, might severely affect HRQoL [3], as demonstrated by the high impact of headache as VS-related symptom on HRQoL in our results.

Our study has several key strengths. Most importantly, in the current era in which HRQoL is highly prioritized, our study is the first to assess patient-preferred outcomes in patients with VS. Moreover, this study combined qualitative and quantitative data on symptoms, management-related side effects, and their impact on HRQoL and daily life. The use of open-ended questions in our questionnaire allowed participants to express themselves freely about living with VS. Other strengths of this study were the high number of included patients and the methodology of independent coding by two of the authors.

Several limitations should, however, also be acknowledged. First of all, even though the large number of participants is a strength, the multiple platforms through which the patients were included form a limitation. While we know Stichting Hoormij send 332 VS patients an email for participation, the call for participation was spread on multiple platforms by both the patient association and patients themselves with an unknown reach. Assuming that most of our included patients were members of Stichting Hoormij, our results might have faced some bias, since members of a VS association are known to report symptoms more actively than patients with VS seen in hospitals [44]. On top of that, 71% of the patients reported to have received active treatment in our study while a large population-based study by Reznitsky et al. shows that 48.4% of 3637 patients were actively treated during a mean 7.33 years of follow-up [45], limiting the generalizability of our results. However, when looking at questionnaire studies in the field of VS, actively treated patients tend to respond more to surveys (70.3–72.5%), comparable to our findings [3, 27, 46]. Yet patients who were actively treated might have had larger tumours upfront with more complaints and a higher burden, though there is a limited relation with tumour size or growth and for example hearing loss [47]. Secondly, our questionnaire did not ask patients about their tumour size since this was not expected to be accurately known. However, this may certainly be an important factor influencing symptoms, management strategy and side effects. Meanwhile, several factors might have contributed to either over- or underestimation of symptoms and self-reported side effects, as some patients mentioned having difficulty distinguishing these two, possibly caused by recall bias with a median time of 8.4 years since treatment. Yet, patients do report differences in symptoms and self-reported side effects, emphasizing the necessity for a distinction between these two. Lastly, data were collected using self-report questionnaires, which may be subject to biases such as social desirability and recall bias. In future research, incorporating qualitative methods, such as interviews or focus groups, could provide a more in-depth understanding of participants' perspectives and experiences, allowing for a richer exploration of their perceptions and insights. Additionally, conducting a pilot phase with a critical reference group to refine the study procedures, materials, and measures in the preparation, organization and reporting phase could further enhance the trustworthiness and validity of the findings [48]. By incorporating these qualitative methods and a pilot phase, future research could build on the strengths of our study and provide a more extensive and comprehensive understanding of the patient-preferred outcomes, symptoms and side effects in patients with VS, enhancing the overall trustworthiness of the findings.

### Recommendations for clinical practice

Several important implications for clinical practice and research follow from this study. First of all, despite the importance of the classic symptoms and endpoints [40, 41], clinicians should focus more on the lesser well-known symptoms and the socio-economic impact patients might encounter and actively ask for these during consultation [43]. Following from the patient remarks (data not shown), there seems to be a dire need for empathy, understanding and public recognition by both healthcare providers and society. As mentioned by multiple patients, adequate (multidisciplinary) follow-up and guidance on the encountered symptoms and side effects, for instance by dedicated specialised VS nurses, would make them feel supported and enable them to cope with their symptoms and side effects. In addition, clinicians could offer involving a social worker, medical psychologist or occupational therapist to support the patients in problems they might encounter in daily life and refer to patient associations for peer support. Following from this, more research should be initiated on the role of energy and fatigue, as also socio-economic consequences in patients with VS in the short and long term. Not only could results of future studies be used to update the PANQOL questionnaire, but these could also be incorporated in clinical practice and possibly positively influence HRQoL.

## Conclusion

Although in management of VS, priorities are shifting from tumour control towards preservation of HRQoL, there are still improvements to make when it comes to patient-centred care for patients with VS. This study, in which the majority of patients with VS was actively treated, not only showed that the well-known classical symptoms hampered patients (e.g. hearing problems, balance disturbances and energy impairments), but it, more importantly, showed that patients encountered participation difficulties in their daily physical and social activities. This ranged from exclusion in social engagements (especially conversations or activities in groups and noisy environments) to being precluded from doing their jobs and societal participation. Healthcare providers should consider these key points when managing patients with VS in consultation, treatment and follow-up.

## Supplementary Information

Below is the link to the electronic supplementary material.

Supplementary file1 (DOCX 36 KB)

## Data Availability

The datasets generated during and analysed during the current study are available from the corresponding author on reasonable request.

## References

[1] Reznitsky M, Petersen M, West N, Stangerup SE, Cayé-Thomasen P (2019). Epidemiology of vestibular schwannomas-prospective 40-year data from an unselected national cohort. Clinical Epidemiology.

[2] Propp JM, McCarthy BJ, Davis FG, Preston-Martin S (2006). Descriptive epidemiology of vestibular schwannomas. Neuro-Oncology.

[3] Carlson ML, Tveiten OV, Driscoll CL, Goplen FK, Neff BA, Pollock BE (2015). Long-term quality of life in patients with vestibular schwannoma: An international multicenter cross-sectional study comparing microsurgery, stereotactic radiosurgery, observation, and nontumor controls. Journal of Neurosurgery.

[4] Soulier G, van Leeuwen BM, Putter H, Jansen JC, Malessy MJA, van Benthem PPG (2017). Quality of life in 807 patients with vestibular schwannoma: Comparing treatment modalities. Otolaryngology.

[5] Gauden A, Weir P, Hawthorne G, Kaye A (2011). Systematic review of quality of life in the management of vestibular schwannoma. Journal of Clinical Neuroscience.

[6] Goldbrunner R, Weller M, Regis J, Lund-Johansen M, Stavrinou P, Reuss D (2020). EANO guideline on the diagnosis and treatment of vestibular schwannoma. Neuro-Oncology.

[7] Carlson ML, Link MJ (2021). Vestibular schwannomas. New England Journal of Medicine.

[8] Neve OM, Jansen JC, van der Mey AGL, Koot RW, de Ridder M, van Benthem PPG (2021). The impact of vestibular schwannoma and its management on employment. European Archives of Oto-rhino-laryngology.

[9] Pruijn IMJ, Kievit W, Hentschel MA, Mulder JJS, Kunst HPM (2020). What determines quality of life in patients with vestibular schwannoma?. Clinical Otolaryngology.

[10] Shaffer BT, Cohen MS, Bigelow DC, Ruckenstein MJ (2010). Validation of a disease-specific quality-of-life instrument for acoustic neuroma: The Penn Acoustic Neuroma Quality-of-Life Scale. The Laryngoscope.

[11] Study protocol for the World Health Organization project to develop a Quality of Life assessment instrument (WHOQOL). *Quality of Life Research, 2*(2):153–159. 8518769

[12] Cella DF (1992). Quality of life: The concept. Journal of Palliative Care.

[13] Papatsoutsos E, Spielmann PM (2018). Self-evaluated quality of life and functional outcomes after microsurgery, stereotactic radiation or observation-only for vestibular schwannoma of the adult patient: A systematic review. Otology & Neurotology.

[14] Montori VM, Brito JP, Murad MH (2013). The optimal practice of evidence-based medicine: Incorporating patient preferences in practice guidelines. JAMA.

[15] Windisch P, Tonn JC, Fürweger C, Ehret F, Wowra B, Kufeld M (2021). Longitudinal changes of quality of life and hearing following radiosurgery for vestibular schwannoma. Cancers (Basel).

[16] Peris-Celda M, Graffeo CS, Perry A, Kleinstern G, Kerezoudis P, Driscoll CLW (2020). Beyond the ABCs: Hearing loss and quality of life in vestibular schwannoma. Mayo Clinic Proceedings.

[17] Santa Maria C, Santa Maria PL, Bulsara V, Jayawardena J, Caldow JD, Png LH (2019). Long-term quality of life in patients with vestibular schwannoma managed with microsurgery. Journal of Laryngology and Otology.

[18] Deberge S, Meyer A, Le Pabic E, Peigne L, Morandi X, Godey B (2018). Quality of life in the management of small vestibular schwannomas: Observation, radiotherapy and microsurgery. Clinical Otolaryngology.

[19] Klersy PC, Arlt F, Hofer M, Meixensberger J (2018). Quality of life in patients with unilateral vestibular schwannoma on wait and see-strategy. Neurological Research.

[20] Foley RW, Maweni RM, Jaafar H, McConn Walsh R, Javadpour M, Rawluk D (2017). The impact of primary treatment strategy on the quality of life in patients with vestibular schwannoma. World Neurosurgery.

[21] Berkowitz O, Han YY, Talbott EO, Iyer AK, Kano H, Kondziolka D (2017). Gamma knife radiosurgery for vestibular schwannomas and quality of life evaluation. Stereotactic and Functional Neurosurgery.

[22] Jufas N, Flanagan S, Biggs N, Chang P, Fagan P (2015). Quality of life in vestibular schwannoma patients managed by surgical or conservative approaches. Otology & Neurotology.

[23] Turel MK, Thakar S, Rajshekhar V (2015). Quality of life following surgery for large and giant vestibular schwannomas: A prospective study. Journal of Neurosurgery.

[24] Scheich M, Ginzkey C, Reuter E, Harnisch W, Ehrmann D, Hagen R (2014). Quality of life after microsurgery for vestibular schwannoma via the middle cranial fossa approach. European Archives of Oto-Rhino-Laryngology.

[25] Wangerid T, Bartek J, Svensson M, Förander P (2014). Long-term quality of life and tumour control following gamma knife radiosurgery for vestibular schwannoma. Acta Neurochirurgica. Supplementum.

[26] McLaughlin EJ, Bigelow DC, Lee JY, Ruckenstein MJ (2015). Quality of life in acoustic neuroma patients. Otology & Neurotology.

[27] Carlson ML, Tveiten ØV, Driscoll CL, Goplen FK, Neff BA, Pollock BE (2015). What drives quality of life in patients with sporadic vestibular schwannoma?. The Laryngoscope.

[28] Miller LE, Brant JA, Naples JG, Bigelow DC, Lee JYK, Ruckenstein MJ (2020). Quality of life in vestibular schwannoma patients: A longitudinal study. Otology & Neurotology.

[29] Müller S, Arnolds J, van Oosterhout A (2010). Decision-making of vestibular schwannoma patients. Acta Neurochirurgica. Supplementum.

[30] Rosahl S, Bohr C, Lell M, Hamm K, Iro H (2017). Diagnostics and therapy of vestibular schwannomas—An interdisciplinary challenge. GMS Current Topics in Otorhinolaryngology - Head and Neck Surgery.

[31] Burla L, Knierim B, Barth J, Liewald K, Duetz M, Abel T (2008). From text to codings: intercoder reliability assessment in qualitative content analysis. Nursing Research.

[32] Sandelowski M (1995). Qualitative analysis: What it is and how to begin. Research in Nursing & Health.

[33] van Hoorn R, Kievit W, Booth A, Mozygemba K, Lysdahl KB, Refolo P (2016). The development of PubMed search strategies for patient preferences for treatment outcomes. BMC Medical Research Methodology.

[34] Say RE, Thomson R (2003). The importance of patient preferences in treatment decisions–challenges for doctors. BMJ.

[35] Myrseth E, Møller P, Wentzel-Larsen T, Goplen F, Lund-Johansen M (2006). Untreated vestibular schwannoma: Vertigo is a powerful predictor for health-related quality of life. Neurosurgery.

[36] Breivik CN, Varughese JK, Wentzel-Larsen T, Vassbotn F, Lund-Johansen M (2012). Conservative management of vestibular schwannoma–A prospective cohort study: Treatment, symptoms, and quality of life. Neurosurgery.

[37] Carlson ML, Lohse CM, Link MJ, Tombers NM, McCaslin DL, Saoji AA (2022). Development and validation of a new disease-specific quality of life instrument for sporadic vestibular schwannoma: The Mayo Clinic Vestibular Schwannoma Quality of Life Index. Journal of Neurosurgery.

[38] Dhayalan D, Lund-Johansen M, Finnkirk M, Tveiten ØV (2019). Fatigue in patients with vestibular schwannoma. Acta Neurochirurgica. Supplementum.

[39] Lloyd SK, Kasbekar AV, Baguley DM, Moffat DA (2010). Audiovestibular factors influencing quality of life in patients with conservatively managed sporadic vestibular schwannoma. Otology & Neurotology.

[40] Foley RW, Shirazi S, Maweni RM, Walsh K, McConn Walsh R, Javadpour M (2017). Signs and symptoms of acoustic neuroma at initial presentation: An exploratory analysis. Cureus.

[41] Blom S, Aarts H, Wever CC, Kunst HPM, Semin GR (2020). Quality of life, social function, emotion, and facial paresis in Dutch vestibular schwannoma patients. Laryngoscope Investigative Otolaryngology.

[42] Preet K, Ong V, Sheppard JP, Udawatta M, Duong C, Romiyo P (2020). Postoperative hearing preservation in patients undergoing retrosigmoid craniotomy for resection of vestibular schwannomas: A systematic review of 2034 patients. Neurosurgery.

[43] Heman-Ackah SE, Golfinos JG, Roland JT (2012). Management of surgical complications and failures in acoustic neuroma surgery. Otolaryngologic Clinics of North America.

[44] Prummer CM, Kerezoudis P, Tombers NM, Peris-Celda M, Link MJ, Carlson ML (2019). Influence of selection bias in survey studies derived from a patient-focused organization: A comparison of response data from a single tertiary care center and the acoustic neuroma association. Otology & Neurotology.

[45] Reznitsky M, Petersen M, West N, Stangerup SE, Cayé-Thomasen P (2021). The natural history of vestibular schwannoma growth-prospective 40-year data from an unselected national cohort. Neuro-Oncology.

[46] Chweya CM, Tombers NM, Lohse CM, Link MJ, Carlson ML (2021). Disease-specific quality of life in vestibular schwannoma: A national cross-sectional study comparing microsurgery, radiosurgery, and observation. Otolaryngology - Head and Neck Surgery.

[47] Patel NS, Huang AE, Dowling EM, Lees KA, Tombers NM, Lohse CM (2020). The influence of vestibular schwannoma tumor volume and growth on hearing loss. Otolaryngology - Head and Neck Surgery.

[48] Elo S, Kääriäinen M, Kanste O, Pölkki T, Utriainen K, Kyngäs H (2014). Qualitative content analysis: A focus on trustworthiness. SAGE Open.

