# Supplementary material for: Patient-preferred outcomes in patients with vestibular schwannoma: a qualitative content analysis of symptoms, side effects and their impact on health-related quality of life
Source: Qual Life Res. 2023 May 31;32(10):2887–97. doi: 10.1007/s11136-023-03433-x (PMC10474211; doi:10.1007/s11136-023-03433-x)
Supplement: Supplementary file 1 — Supplementary file1 (DOCX 36 KB) [file 11136_2023_3433_MOESM1_ESM.docx]

**Supplementary Table 1: Patient-preferred outcomes by VS-patients**

| Categories | N = 231 |  |
| --- | --- | --- |
|  | Participants, n (%) | Symptom expressions (% of total citations) |
|  |  |  |
|  |  |  |
| **Hearing preservation** | **141 (61%)** | 141 |
| **Balance preservation** | **89 (38.5%)** | 89 |
| Dizziness (vertigo) |  | 14 (15.7%) |
| **Tinnitus reduction** | **80 (34.6%)** | 80 |
| **Restored Energy** | **50 (21.6%)** | 50 |
| Energy, other |  | 36 (72%) |
| Fatigue |  | 14 (28%) |
| **Facial issues reduction** | **42 (18.2%)** | 45 |
| Facial nerve |  | 34 (75.6%) |
| Trigeminal nerve |  | 6 (13.3%) |
| Smell and taste |  | 5 (11.1%) |
| **Emotional health improvement** | **34 (14.8%)** | 36 |
| Happiness |  | 16 (44.4%) |
| Feeling safe and secure |  | 12 (33.3%) |
| Anxiety/Stress |  | 5 (13.9%) |
| Emotional health, other |  | 2 (5.6%) |
| Independence |  | 1 (2.8%) |
| **Pain reduction** | **22 (9.5%)** | 22 |
| Headache |  | 20 (90.9%) |
| Pain, other (backache and neckaches) | | 2 (9.1%) |
| **Treatment related improvement** | **11 (4.8%)** | 11 |
| Worries for side effects |  | 7 (63.6%) |
| Length of recovery |  | 4 (36.4%) |
| **Cognitive function improvement** | **11 (4.8%)** | 12 |
| Better concentration/focus | | 6 (50%) |
| Cognitive function, other |  | 4 (33.3%) |
| Sleeping-ability |  | 1 (8.3%) |
| Memory enhancement |  | 1 (8.3%) |
| **Tumor control** | **10 (4.3%)** | 10 |
| **Other improvements** | **9 (3.8%)** | 9 |
| **Social health improvement** | **8 (3.5%)** | 10 |
| Social inclusion |  | 7 (70%) |
| Participation in society |  | 3 (30.0%) |
| **Maintaining HRQoL** | **8 (3.5%)** | 8 |
| **Hyperacusis reduction** | **2 (0.87%)** | 2 |
| **Ability to work** | **2 (0.87%)** | 2 |
| **Sports improvement** | **1 (0.43%)** | 1 |

*VS* vestibular schwannoma, *n* number, *HRQoL* health-related quality of life

**Supplementary Table 2:** **Experienced management-related side effects, their impact and frequencies.**

| Categories | N = 129^a^ |  |  |  |  |  |  |
| --- | --- | --- | --- | --- | --- | --- | --- |
|  | Participants, n (%) | Symptom expressions (% of total citations) | Mean impact on HRQoL (SD) | Frequency (%) | | | |
|  |  |  |  |  |  |  |  |
|  |  |  |  | Continuous | Often | Sometimes | Rarely |
| **Hearing** | **82 (63.6%)** | 85 |  |  |  |  |  |
| Deafness |  | 57 (67.1%) | 7.5 (2.2) | 61.4% | 28.1% | 7.0% | 1.8% |
| Hearing loss |  | 23 (27.1%) | 7.4 (1.5) | 56.5% | 30.4% | 13.0% | 0.0% |
| Hyperacusis |  | 4 (4.7%) | 8 (0.8) | 75.0% | 0.0% | 25.0% | 0.0% |
| Sound localization |  | 2 (2.4%) | 8 (NA) | 50.0% | 50.0% | 0.0% | 0.0% |
| **Balance** | **63 (48.8%)** | 65 |  |  |  |  |  |
| Imbalance |  | 49 (75.4%) | 6.4 (2.2) | 34.7% | 36.7% | 26.5% | 2.0% |
| Dizziness (vertigo) |  | 13 (20.0%) | 6.7 (2.2) | 23.1% | 30.8% | 38.5% | 7.7% |
| Nausea |  | 2 (3.1%) | 7.5 (NA) | 0.0% | 100.0% | 0.0% | 0.0 |
| Light-headed |  | 1 (1.5%) | 2 (NA) | 0.0% | 0.0% | 0.0% | 100.0% |
| **Face** | **51 (39.5%)** | 61 |  |  |  |  |  |
| Facial nerve |  | 35 (57.4%) | 7 (2.2) | 54.3% | 28.6% | 11% | 5.7% |
| Eye problems |  | 13 (21.3%) | 7.2 (2.7) | 46.2% | 15.4% | 23% | 15.4% |
| Trigeminal nerve |  | 12 (19.7%) | 6 (2.7) | 16.7% | 58.3% | 25% | 0.0% |
| Smell and taste |  | 1 (1.6%) | 7 (NA) | 100.0% | 0.0% | 0.0% | 0.0% |
| **Energy** | **42 (32.6%)** | 42 |  |  |  |  |  |
| Fatigue |  | 37 (88.1%) | 7.5 (1.8) | 27.0% | 56.8% | 13.5% | 2.7% |
| Other |  | 5 (11.9%) | 7.8 (1.5) | 60.0% | 40.0% | 0.0% | 0.0% |
| **Tinnitus** | **37 (28.7%)** | 37 | 7 (2.1) | 66.7% | 11.9% | 19.0% | 2.4% |
| **Cognitive impairment** | **25 (19.4%)** | 32 |  |  |  |  |  |
| Concentration |  | 15 (46.9%) | 6.9 (1.4) | 26.7% | 53.3% | 20.0% | 0.0% |
| Overstimulated/chaotic |  | 5 (15.6%) | 6.6 (1.1) | 0.0% | 100% | 0.0% | 0.0% |
| Memory impairment |  | 5 (15.2%) | 7 (1.4) | 20.0% | 20.0% | 60.0% | 0.0% |
| Other |  | 5 (15.6%) | 6.8 (3.0) | 40% | 40% | 0% | 20% |
| Sleeping |  | 2 (6.1%) | 6 (NA) | 0.0% | 50.0% | 0.0% | 50.0% |
| **Pain** | **24 (18.6%)** | 27 |  |  |  |  |  |
| Headache |  | 23 (85.2%) | 7 (2.4) | 26.1% | 34.8% | 30.4% | 8.7% |
| Not specified |  | 3 (11.1%) | 7.3 (2.1) | 33.3% | 66.7% | 0.0% | 0.0% |
| Neck pain |  | 1 (3.7%) | 9 (NA) | 100.0% | 0.0% | 0.0% | 0.0% |
| **Post treatment, other** | **16 (12.4%)** | 16 | 7.4 (1.9) | 6.3% | 18.8% | 38.0% | 37.5% |
| **Psychological impairment** | **12 (9.3%)** | 13 |  |  |  |  |  |
| Anxiety |  | 3 (23.1%) | 3(1) | 0.0% | 33.3% | 33.3% | 33.3% |
| Insecurity |  | 3 (23.1%) | 7 (1) | 33.3% | 66.7% | 0.0% | 0.0% |
| Emotional |  | 2 (15.4%) | 2.5 (NA) | 50% | 0.0% | 50% | 0.0% |
| Frustration and tension |  | 2 (15.4%) | 7 (NA) | 0.0% | 50.0% | 50.0% | 0.0% |
| Sadness/Depression |  | 2 (15.4%) | 2.5 (NA) | 0.0% | 0.0% | 0.0% | 100.0% |
| Other |  | 1 (7.7%) | 3 (NA) | 0.0% | 0.0% | 100% | 0.0% |
| **Socioeconomical** | **10 (7.8%)** | 11 |  |  |  |  |  |
| Social interaction |  | 5 (45.5%) | 8 (0.7) | 20.0% | 80.0% | 0.0% | 0.0% |
| Leisure time (hobby, sports) | | 3 (27.3%) | 8.3 (1.2) | 66.7% | 0.0% | 33.3% | 0.0% |
| Avoiding busy places | | 2 (18.2%) | 7 (NA) | 0.0% | 50.0% | 50.0% | 0.0% |
| Job (loss/not functioning) | | 1 (9.1%) | 9 (NA) | 100% | 0.0% | 0.0% | 0.0% |
| **Post treatment symptoms** | **5 (3.9%)** | 5 |  |  |  |  |  |
| Wound infection |  | 1 (20%) | 4 (NA) | 100% | 0.0% | 0.0% | 0.0% |
| CVA |  | 1 (20%) | 9 (NA) | 100% | 0.0% | 0.0% | 0.0% |
| Trombosis |  | 1 (20%) | 3 (NA) | 0.0% | 0.0% | 100% | 0.0% |
| CSF leakage |  | 1 (20%) | 5 (NA) | 0.0% | 0.0% | 100% | 0.0% |
| Hallucinations |  | 1 (20%) | 4 (NA) | 0.0% | 0.0% | 0.0% | 100% |

^a^ treated patients n= 125, patients in wait and scan protocol n= 4

*n* number, HRQoL health-related quality of life, *SD* standard deviation, *NA* not applicable, *CVA* cerebrovascular accident, *CSF* cerebrospinal fluid

| Categories | N = 83^a^ |  |
| --- | --- | --- |
|  | Participants, n (%) | Symptom expressions (% of total citations) |
|  |  |  |
|  |  |  |
| **Social engagements** | **72 (86.7%)** | 105 |
| Social contacts/activities |  | 57 (54.3%) |
| Group conversations |  | 48 (45.7%) |
| **Work and tasks** | **50 (60.2%)** | 65 |
| Job |  | 41 (63.1%) |
| Domestic chores |  | 17 (26.2%) |
| Other |  | 5 (7.7%) |
| Education |  | 2 (3.1%) |
| **(Avoiding) busy place** | **47 (56.6%)** | 47 |
| **Sports** | **29 (34.9%)** | 30 |
| General, sports |  | 27 (90.0%) |
| Hiking |  | 2 (6.7%) |
| Running |  | 1 (3.3%) |
| **Traffic participation** | **22 (26.5%)** | 24 |
| Cycling |  | 15 (62.5%) |
| Car driving |  | 4 (16.7%) |
| Walking |  | 2 (8.3%) |
| Motor-cycling |  | 1 (4.2%) |
| Avoidence of public places | | 1 (4.2%) |
| Other |  | 1 (4.2%) |
| **Leisure activities** | **19 (22.9%)** | 22 |
| Hobby |  | 14 (63.6%) |
| Enjoying music |  | 8 (36.4%) |
| **Cultural activities** | **13(15.7%)** | 14 |
| Theatre/Cinema |  | 9 (64.3%) |
| Concerts/Festivals |  | 4 (28.6%) |
| Other |  | 1 (7.1%) |
| **Other** | **13 (15.7%)** | 13 |
| **Personal care** | **4 (4.8%)** | 4 |
| **Emotional expressions** | **4 (4.8%)** | 4 |

**Supplementary Table 3:** **Impact of management-related side effects on daily life of VS patients (N=83).**

^a^ treated patients n= 83, patients in wait and scan protocol n= 0.
